# Effects and Mechanisms of Luteolin, a Plant-Based Flavonoid, in the Prevention of Cancers via Modulation of Inflammation and Cell Signaling Molecules

**DOI:** 10.3390/molecules29051093

**Published:** 2024-02-29

**Authors:** Saleh A. Almatroodi, Ahmad Almatroudi, Hajed Obaid A. Alharbi, Amjad Ali Khan, Arshad Husain Rahmani

**Affiliations:** 1 Department of Medical Laboratories, College of Applied Medical Sciences, Qassim University, Buraydah 51452, Saudi Arabia; smtrody@qu.edu.sa (S.A.A.);; 2 Department of Basic Health Sciences, College of Applied Medical Sciences, Qassim University, Buraydah 51452, Saudi Arabia; akhan@qu.edu.sa

**Keywords:** luteolin, oxidative stress, inflammation, apoptosis, signal transduction pathway, cancer therapy

## Abstract

Luteolin, a flavonoid, is mainly found in various vegetables and fruits, including carrots, cabbages, onions, parsley, apples, broccoli, and peppers. Extensive research in vivo and in vitro has been performed to explore its role in disease prevention and treatment. Moreover, this compound possesses the ability to combat cancer by modulating cell-signaling pathways across various types of cancer. The studies have confirmed that luteolin can inhibit cancer-cell survival and proliferation, angiogenesis, invasion, metastasis, mTOR/PI3K/Akt, STAT3, Wnt/β-catenin, and cell-cycle arrest, and induce apoptosis. Further, scientific evidence describes that this compound plays a vital role in the up/down-regulation of microRNAs (miRNAs) in cancer therapy. This review aims to outline the anti-cancer mechanisms of this compound and its molecular targets. However, a knowledge gap remains regarding the studies on its safety and efficacy and clinical trials. Therefore, it is essential to conduct more research based on safety, efficacy, and clinical trials to explore the beneficial role of this compound in disease management, including cancer.

## 1. Introduction

Cancer is a severe health issue that affects countless individuals around the world. Unfortunately, the incidence and mortality rates of cancer have been increasing in recent years. Many symptoms can be attributed to the development and progression of cancer, leading to disruptions in various cellular metabolic pathways [1]. Different treatment options are utilized to combat cancer, such as chemotherapy, radiotherapy, and surgical removal. Unfortunately, the effectiveness of these therapies is often reduced due to cell resistance [2]. In addition to the well-known side effects of chemotherapy, radiotherapy can cause vomiting, mouth ulcers, fatigue, constipation, and hair loss. As a result, health researchers are focusing on developing safe and effective treatment options that can help overcome these side effects and improve the quality of life for cancer patients. Plant-based drug development has been gaining traction as a potential solution for creating safe and effective anti-tumor drugs [3,4]. Researchers can develop highly targeted and potent treatments by leveraging the complete knowledge of the synergistic relationship between several components of anti-tumor herbs [5,6,7]. Some phytochemicals have chemosensitizing potential and can help reduce the development of toxic side effects caused by chemotherapy.

Luteolin is a flavone chiefly found in vegetables and fruits [8]. Moreover, it is isolated as a yellow crystalline compound with the chemical formula C_15_H_10_O_6_, a tetrahydroxyflavone in which the four hydroxy groups are present at positions 3′, 4′, 5, and 7 [9]. Its role in disease management by modulating various biological activities has been discussed. This compound has been proven to have antioxidant, anti-inflammatory, anti-apoptotic, and hepatoprotective properties [10,11]. The role of luteolin in cancer has been firmly established through extensive in vivo and in vitro studies that highlight its ability to modulate cell-signaling pathways. This review delves into the potential of luteolin as an anticancer agent by analyzing its ability to modulate various cell-signaling pathways.

## 2. Chemical Structure and Sources of Luteolin

**Figure 1:**
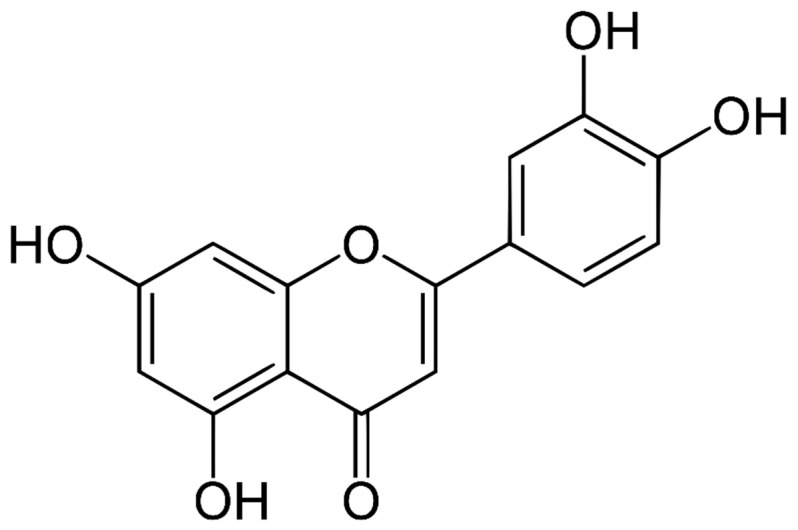
Luteolin chemical structure.

Luteolin (3′,4′,5,7-tetrahydroxy flavone) is a phenolic phytochemical belonging to the flavone class of flavonoids (Figure 1) and chiefly found in vegetables and fruits [8] as well as in a diversity of plant species. Luteolin or its derivatives can be found in over 300 plant species, making it a well-known and widely distributed compound among plants [12].

**Figure 2:**
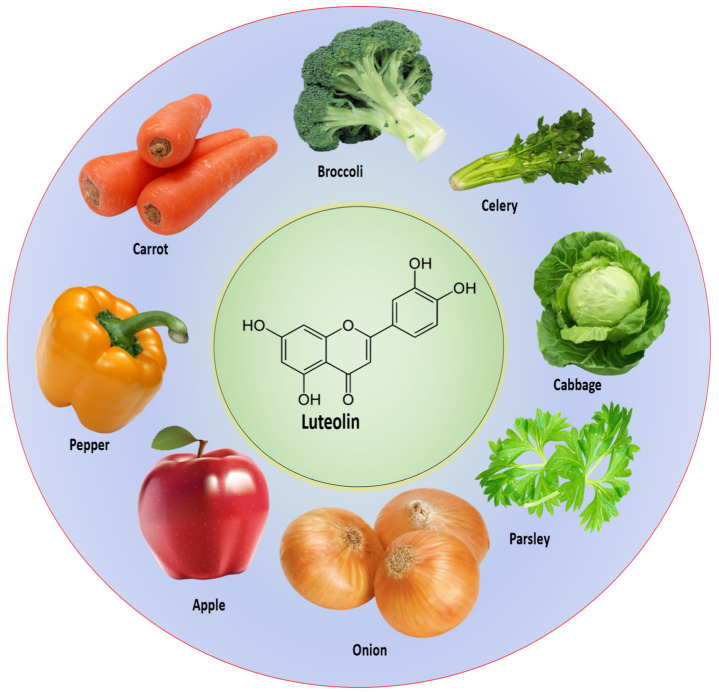
Luteolin sources in different plants [14].

Vegetables and fruits are rich sources of a compound called luteolin. Some foods abundant in luteolin include carrots, cabbages, onion leaves, celery, parsley, broccoli, peppers, apple skins, and even chrysanthemum flowers [13,14,15,16]. Moreover, vegetables are rich sources of luteolin, also present in flowers. Table 1 [14] (Figure 2) describes the luteolin sources in different plants.

## 3. The Role of Luteolin in Cancer Prevention through Modulation of Cell Signaling Pathways

**Figure 3:**
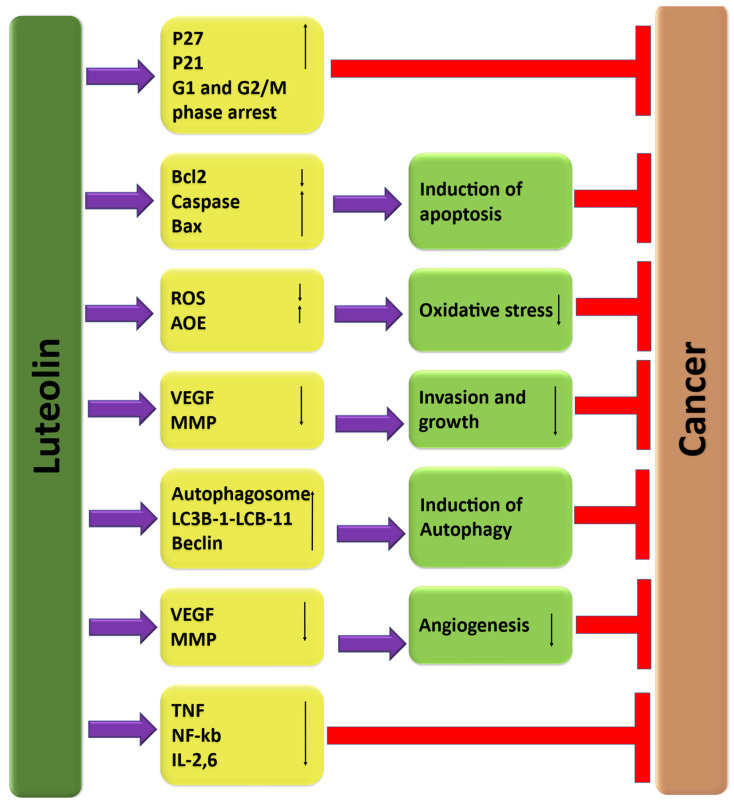
Luteolin mechanisms in cancer management through modulation of apoptosis, angiogenesis, oxidative stress, and autophagy. The upward pointing arrow signifies upregulation and the downward pointing arrow signifies downregulation. The block arrow signifies the inhibition.

It is interesting to note that luteolin, a type of flavonoid commonly found in fruits and vegetables, has been shown to have potential in managing cancer. It was reported that luteolin could be crucial in cancer management by targeting various cancer development and progression mechanisms. These mechanisms include regulating inflammation, inducing cell-cycle arrest, promoting apoptosis, inhibiting angiogenesis, and modulating the PI3/Akt, STAT3, EFGR, and autophagy pathways (Figure 3). The extensive role that luteolin plays in cancer management through these different mechanisms has been well documented in scientific research (Table 2), and it is summarized below.

### 3.1. Inflammation

Almost 25% of all human malignancies are associated with chronic inflammation, and viral and bacterial infections [17]. During tumor development, inflammatory mediators such as reactive oxygen species, cytokines, and reactive nitrogen species derived from tumor-infiltrating immune cells can initiate epigenetic changes in tumor suppressor genes, ultimately leading to a pre-malignant state [18]. Preventing the production or role of these inflammatory cytokines and mediators is believed to be a crucial step in regulating inflammation and reducing the risk of cancer development [19].

Luteolin has been found to possess substantial anti-inflammatory properties by inhibiting the production of inflammatory cytokines and mediators. Luteolin has been found to have the potential to partially prevent the production of nitric oxide (NO) and the function and expression of inducible nitric oxide synthase (iNOS) [20]. Such prevention was associated with the capability of luteolin to control ROS because NO is a labile radical object [21].

Liver cancer in BALB/c mice was induced through the administration of diethylnitrosamine (DN) in their drinking water. So, to treat DN-induced liver cancer in mice, 20 µg/kg of body weight luteolin was administered intraperitoneally every alternate day. According to the study, significant differences were observed in the levels of AST (aspartate aminotransferase) and ALT (alanine aminotransferase) in the plasma of mice with DN-induced liver cancer. Specifically, these levels were 109.8% and 148.3%, respectively. Similarly, in the liver tissue, the levels of AST and ALT were 55.5% and 61.3%, respectively. However, all these alterations returned to normal levels during treatment with luteolin. This suggests that luteolin may effectively combat the liver damage caused by DN induction. In addition to normalizing AST and ALT levels, LUT also appeared to reduce the levels of glutathione and the inflammatory cytokines interleukin-2 and interferon-γ in either the plasma or liver tissue. The study’s findings suggest that luteolin may be a valuable treatment for liver cancer [22].

The study investigated the effects of luteolin on inflammation and oxidative stress-related cytokines. The results showed that luteolin significantly reduced the levels of various cytokines such as IL-6, IFN-β, TNF-α, and IL-1 β compared to the model group. Additionally, it increased the levels of IL-2 and IL-10 compared to the model group. The results showed that in luteolin-treated groups, the expression levels of NLRP3, IL-1 β caspase-1 protein, and mRNA were meaningfully blocked compared to the model group. Moreover, the model group displayed increased activation of the NLRP3/IL-1 β signal axis compared to the vehicle group, but luteolin reduced this activation in a dose-dependent manner. It seems that these findings demonstrate that luteolin may be able to inhibit tumor cell growth by suppressing the NLRP3/IL-1 β signal axis [23].

The colitis-based study reported that luteolin increased serum and intestinal cytokine levels. Moreover, luteolin meaningfully decreased the expression of HMGB1-TLR-NF-κB signaling pathway protein. This compound significantly reduced and improved DSS-induced colitis, and the mechanism was associated with controlling the intestinal HMGB1-TLR-NF-κB signaling pathway [24]. Luteolin suppressed TNF-α-induced interleukin-8 production in a dose-dependent way. Luteolin has the potential to inhibit TNF-α-induced IL-8 production in intestinal epithelial cells. This is due to its ability to block the phosphorylation of MAPKs, IκB degradation, and NF-κB activation [25]. These findings further suggest that luteolin could have potential therapeutic uses in treating inflammatory bowel diseases [25].

### 3.2. Angiogenesis

It appears that the angiogenic process is triggered in response to hypoxia, which is caused by a lack of nutrients. This leads to an increase in the expression of inflammatory signals and cytokines, which then recruit vascular cells to facilitate the development of a tumor vessel plexus [26,27]. It is known that initiation of blood vessel formation is brought about when pro-angiogenic signaling is dominant. This process is commonly referred to as the “angiogenic switch” in tumors [28]. In this scenario, the concept of angio-prevention arises, mentioning cancer prevention via inhibition and/or stabilization of tumor angiogenesis [29,30].

Natural products can play a role in preventing tumors by inhibiting angiogenesis. It has been proven that they can inhibit the formation of new blood vessels that tumors need to grow and spread. Luteolin prevented tumor growth and angiogenesis in a murine xenograft model. Additionally, luteolin prevented vascular endothelial growth factor (VEGF)-induced in vivo angiogenesis. Further, luteolin inhibited vascular endothelial growth factor-induced survival and proliferation of human umbilical vein endothelial cells. Luteolin inhibited vascular endothelial growth factor-induced phosphatidylinositol 3′-kinase activity in HUVECs, and this prevention was vital for both the antimitotic effects and antisurvival of the compound. Indeed, this compound eliminated VEGF-induced activation of Akt, a downstream target of PI3K conveying both survival and mitotic downstream signals [31]. Luteolin decreased the expression and secretion of VEGF in human choroidal melanoma cells (C918 and OCM-1). In this study, after conducting the Western blot assay, VEGF protein expression was lower in each treated group of the two cell lines compared to the control group. According to the ELISA results, it has been found that the secretion of VEGF by cells decreased when they were exposed to a concentration of 10 to 15 µmol/L luteolin [32].

HUVECs exhibited a high rate of proliferation when stimulated with VEGF. Luteolin treatment meaningfully inhibited VEGF-induced proliferation of HUVECs. Further, HUVECs migrated into a precise area when stimulated with VEGF. VEGF-induced migration of endothelial cells was inhibited by luteolin in a dose-dependent manner. Nearly fully inhibited endothelial cell migration was seen at 40 µmol/L of luteolin compared to 0 h incubation. Additionally, treatment with luteolin (20 and 40 µmol/egg) considerably decreased the vascular density that established the anti-angiogenic activity of luteolin via ex vivo assay. The matrigel plug assay effectively checked the anti-angiogenesis effects of luteolin ex vivo. Luteolin inhibited VEGF-induced angiogenesis, showing promising anti-angiogenic effects [33]. 

### 3.3. Apoptosis

**Figure 4:**
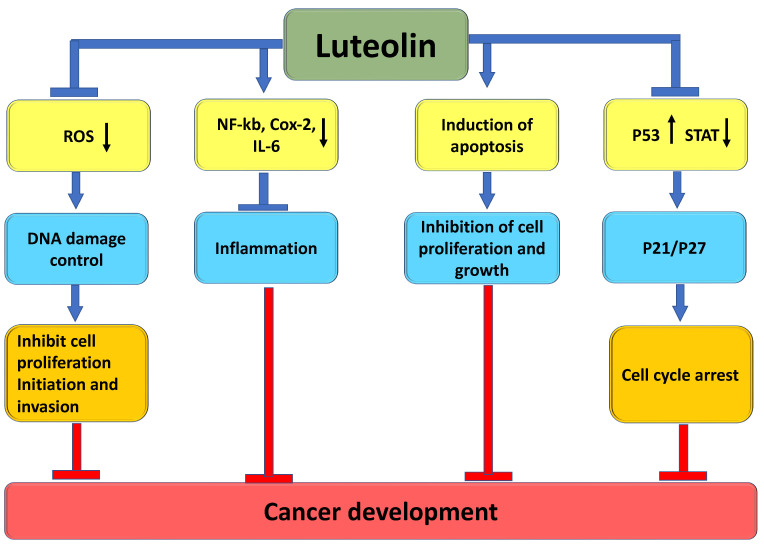
Luteolin has a potential role in cancer management through several mechanisms. The upward pointing arrow signifies upregulation and the downward pointing arrow signifies downregulation. The block arrow signifies the inhibition.

Apoptosis is a fascinating process in the human body that involves programmed cell death. It is tightly regulated at the gene level, which allows for the effective and ordered removal of damaged cells [34], including those occurring following DNA damage or during development. Alteration of this death process is linked with unrestricted cell proliferation and development, as well as progression of cancer and cancer resistance to drug therapies [35,36]. Current treatments, including chemotherapy [37] and radiotherapy [38], can induce cell death via several molecular mechanisms, though they have drawbacks, such as chemo- and radio-resistance [39]. Luteolin may play a role in cancer management through the induction of apoptosis (Figure 4), a process that causes cancer cells to die. Treatment of cancer using natural compounds and their semi-synthetic analogs, both in vivo and in vitro, displays auspicious results against several malignancies [40,41]. Annexin V-FITC and PI were used to detect early and late apoptosis. According to the research findings, the percentage of viable cells decreased significantly from 90.5% in the control cells to 65.4% and 47.9% when treated with luteolin at concentrations of 10 and 20 µM, respectively. Moreover, there was a marked increase in early apoptotic cells from 2.89% in control samples to 9.75% and 12.4% when treated with luteolin at concentrations of 10 and 20 µM [42]. To examine whether luteolin causes cytotoxicity via inducing apoptosis, a study was performed. Results showed that the nuclei were stained with Hoechst 33342 to determine whether luteolin elicits cytotoxicity by inducing apoptosis. The microscopy assessment discovered that the nuclei were intact in the control cells, while luteolin induced nuclear fragmentation, which is a feature of apoptosis, in the colon cancer cells in a dose-dependent manner. Furthermore, the study found that luteolin upregulated the expression of the apoptotic protein Bax, active caspase-9, and active caspase-3, whereas it downregulated the expression of the anti-apoptotic protein Bcl-2 in a dose- and time-dependent way [43]. A glioma cells-based study was performed to check the morphological and biochemical features of apoptosis after treatment of cancer cells with different concentrations of luteolin. As per the examination under fluorescence microscopy, apoptosis bodies and karyorrhexis meaningfully increased as compared to the control group. The outcomes designated that luteolin caused the morphological changes in these cancer cells. According to flow cytometric analysis, after treatment with luteolin, there was a significant increase in the percentages of early and late apoptotic cells. The exposure to luteolin for 24 h led to an important rise in both early and late apoptotic cells. Precisely, the percentage of early apoptotic cells increased from 3.09% to 21.59%, whereas the percentage of late apoptotic cells also showed a clear rise in response to increased luteolin concentration [44].

### 3.4. Autophagy

Autophagy is a natural cellular mechanism that helps eliminate and break down misfolded proteins and damaged organelles. It plays a critical role in adapting to various conditions such as starvation, cell death, development, and even the suppression of tumors [45,46]. Once malignant cancers are entirely developed, increased autophagy enables tumor cell survival and growth [47,48]. Therefore, in premalignant lesions, increasing autophagy might stop cancer [49]. The use of luteolin in treating Huh7 cells has revealed promising results. The study found that luteolin treatment could improve the expression of LC3-II and decrease the expression of p62, which indicates DR5 upregulation. Moreover, TEM analysis exhibited the presence of autophagic and vacant vacuoles in the luteolin-treated cells, demonstrating that luteolin can trigger autophagy in Huh7 cells. Furthermore, the study also found that co-treatment with luteolin, TRAIL, and chloroquine inhibited the increase in cleaved caspase-8 and cleaved caspase-3 induced by luteolin and TRAIL. These findings suggest that the luteolin-induced enhancement of TRAIL-initiated apoptosis and the promotion of the autophagic flux were both suppressed by chloroquine [50]. In RPMI 8826 cells, treatment with luteolin at a concentration of 20µmol/L for 48 h caused an important increase in the expression of cleaved caspase 3 as well as LC3 Ⅱ/Ⅰ. However, when chloroquine was given simultaneously, the expression levels of both cleaved caspase 3 and LC3 Ⅱ/Ⅰ meaningfully decreased [51]. The findings of a recent study on hepatocellular carcinoma suggest that luteolin has the potential to be an effective anti-cancer agent. The study found that luteolin could significantly reduce the viability of cancer cells in a time- and dose-dependent manner. In addition, luteolin was discovered to induce significant G0/G1-phase arrest, a process that can prevent cancer cells from dividing and growing. Additionally, luteolin has been found to have some interesting effects on intracellular autophagosomes. According to research, it can enhance the number of these structures, promote the conversion of LC3B-I to LC3B-II, and increase the expression of Beclin 1. Lastly, co-treatment with the autophagy inhibitor chloroquine can reduce the effects of luteolin on cell apoptosis [52]. Luteolin suppresses autophagy but then increases apoptosis initiated by cisplatin and promotes sensitivity to cisplatin via suppressing the expression of RARP1 in ovarian cancer [53]. Luteolin induced autophagy in osteosarcoma and acted as an enhancer to sensitize doxorubicin (DOX)-initiated autophagy signaling. The co-treatment of luteolin and doxorubicin significantly reduces the growth of osteosarcoma cells, showing synergistic cytotoxicity. These results designate that luteolin, combined with doxorubicin, may be an innovative approach for treating human osteosarcoma [54]. Another study investigated the potential of luteolin as an anti-cancer agent in NCI-human lung carcinoma cells. The study demonstrated that luteolin triggers apoptotic cell death in NCI-human lung carcinoma cells by modulating the extrinsic and intrinsic pathways. Moreover, preventing autophagy via bafilomycin A1 drops apoptotic cell death, demonstrating that luteolin-induced autophagy functions as a cell-death mechanism [55]. 

### 3.5. Cell Cycle

Cell-cycle regulation is a critical process that plays a key role in the proliferation, metastasis, and recurrence of tumor cells [56]. Understanding the complex mechanisms that govern cell-cycle progression is essential for developing effective cancer treatments. In cancer treatment, cell-cycle regulation is primarily focused on regulating the expression of associated genes and the action of intracellular proteins, enzymes, or signal factors involved in the control of the cell cycle [57,58,59]. Cyclin-dependent kinases and their cyclin subunits control cell-cycle progression timely at the two checkpoints, G1/S and G2/M [60]. The altered cell cycle has been noticed in different types of cancers. Luteolin may be in cancer management by inducing cell-cycle arrest in cancer cells (Figure 4). To determine if luteolin can hinder the growth of colon cancer HT-29 cells, researchers examined its role in reducing the number of viable cells over a three-day treatment period. The study found that luteolin decreased the number of viable HT-29 cells in a dose-dependent manner, with a significant 83 ± 2% decrease in cell number observed within 72 h of adding luteolin at a concentration of 60 μmol/L. These studies indicate that luteolin promotes G1 and G2/M arrest in HT-29 cells. Additionally, HT-29 cells were incubated for 2 h with different doses of luteolin, with a dose-dependent decrease in cyclin D1 levels and an 86% decrease in protein levels in cells treated with luteolin (60 μmol/L) [61]. A study based on breast cancer was performed to evaluate whether luteolin-mediated growth suppression was due to cell-cycle involvement. Following treatment of MDA-MB-231 cells with luteolin (10 and 30 µM) for 24 h, the treated cells were subjected to analysis of cell-cycle progression. The results demonstrated that although there were substantial differences between the 10-µM luteolin-treated group and the control group, subsequent 30-µM luteolin-treated MDA-MB-231 cells showed a 1.21-fold increase in the number of cells in the S-phase, in comparison with the control [62]. To explain whether luteolin shows an anticancer effect in breast cancer, MCF-7 breast cancer cells were treated with luteolin, and apoptosis was examined. Luteolin prevented growth via perturbation of cell-cycle progression at the sub-G1 as well as G1 phases in MCF-7 cells. Additionally, luteolin increased the expression of death receptors, including DR5, and activated caspase cascades. Based on the findings, it was reported that luteolin can cause cell-cycle arrest and trigger apoptosis by activating both the extrinsic and intrinsic pathways [63]. To investigate the potential antiproliferative effects of luteolin, a study was performed, and flow cytometry analysis indicated that treatment with luteolin led to a significant increase in the proportion of cells in the S-phase of the cell cycle, as well as a concomitant decrease in the proportion of cells in the G1-phase and suggesting that luteolin induced cell-cycle arrest at the S-phase. [64].

### 3.6. Signal Transducer and Activator of Transcription 3 (STAT3)

Signal transducer and activator of transcription 3 (STAT3) is currently recognized as an oncogene, and in nearly 70% of cancers, abnormal regulation of STAT3 has been confirmed [65,66]. Inhibition of STAT3 is a critical step in the control of tumor development and progression. Targeting STAT3 can positively inhibit tumor growth in HNSCC cancer cells, which confirms the belief that STAT3 is an oncogene. Downregulating Bcl-xL expression to enhance apoptosis of cancer cells is a promising strategy for cancer treatment [67]. Drug-resistant gastric cancer-cell lines such as SGC7901/DDP, BGC823, and HGC27 exhibit higher activation of STAT3 compared to drug-sensitive GC cell lines. Based on the evidence presented, it is suggested that luteolin may have the potential to inhibit STAT3 signaling and thus possess antitumor effects in gastric cancer cells. According to the findings, the treatment with luteolin significantly inhibited the phosphorylation of STAT3 in SGC7901/DDP cells. The luteolin treatment significantly inhibited the phosphorylation of STAT3 but did not affect the phosphorylation of Erk, STAT1, and Akt. The activity of luteolin was found to be dependent on the dose and was reported in both HGC27 and SGC7901/DDP cells [68]. The study revealed that luteolin had an inhibitory effect on the phosphorylation of STAT3 and its upstream kinase, Src. Additionally, it was found that luteolin could accelerate the degradation of STAT3 through the ubiquitin-proteasome pathway. Moreover, luteolin was also noticed to downregulate the expression of genes that are targeted by STAT3 as well as are involved in cell survival and invasion in melanoma cells. Animal studies showed that prophylactic administration of luteolin controlled melanoma growth as well as Src/STAT3 signaling in both A375 and B16F10 melanoma-bearing mice. Furthermore, luteolin’s anti-melanoma potential was reduced by STAT3 over-activation in A375 cells. The findings of the study propose that luteolin is capable of inhibiting STAT3 signaling in melanoma cells by suppressing its activation and promoting the degradation of STAT3 protein [69]. It is interesting to note that the effects of luteolin treatment on STAT3 activity were observed in SW1990 and PANC-1 cells. The researchers examined the phosphorylated STAT3 (p-STAT3) level after treating the cells with various concentrations of luteolin. It was detected that luteolin could deactivate p-STAT3, as its levels reduced more meaningfully than EMT-related markers and MMPs with an increase in luteolin concentration. The effects of luteolin on STAT3 activity in SW1990 and PANC-1 cells were noticed. Notably, when luteolin concentrations reached 80 μM, p-STAT3 was nearly undetectable in both cell types [70]. Overexpression of Hsp90 plays a vital role in stopping the degradation of Tyr705-phosphorylated STAT3 initiated by luteolin. Moreover, luteolin has also been effective in reducing the levels of some other Hsp90 interacting proteins. Luteolin can induce cancer-cell apoptosis by promoting Tyr705- and Ser727-phosphorylated STAT3 degradation. This mechanism is achieved through the interaction of luteolin with Hsp90, which causes the degradation of STAT3 [71].

### 3.7. PI3K/Akt Pathway

The PI3K/Akt pathway can be abnormally activated via several mechanisms, such as different genomic alterations, including mutations of phosphatase and tensin homolog (PTEN), PIK3CA, TSC1, Akt, and mechanistic target of rapamycin (mTOR) [72]. The PI3K/AKT/mTOR signaling pathway has been extensively researched and has been proven vital for RT resistance in several cancer types [73,74,75]. Luteolin has been observed to inhibit the proliferation of tamoxifen-resistant ER-positive breast cancer cells and induce apoptosis [76]. The research aimed to examine the effects of luteolin on the regulation of epithelial-mesenchymal transition (EMT) in lung cancer cells and the mechanisms involved. Specifically, the experiment utilized lung adenocarcinoma cells and exposed them to luteolin, followed by administration of TGF-β1. The experiment results showed that pretreatment of luteolin successfully inhibited the morphological changes induced by TGF-β1, as well as the downregulation of E-cadherin in the A549 cells. The activation of the PI3K-Akt-IκBa-NF-κB-Snail pathway, which can lead to a decrease in E-cadherin, was reduced when luteolin was used as a pretreatment in response to TGF-β1 [77]. The study is focused on exploring the potential role of PI3K and MAPK signaling pathways in regulating apoptosis in a specific type of cell (mentioned as BGC-823) upon exposure to luteolin. The study’s findings suggest that luteolin may downregulate the phosphorylation of extracellular signal-regulated kinase in the MAPK signaling pathway and inhibit the phosphorylation of AKT and PI3K. The chief elements of the PI3K, as well as MAPK signaling pathways, were assessed by Western blotting in BGC-823 cells exposed to luteolin with different concentrations (0, 20, 40, and 60 µM) for 48 h. Results reported that luteolin reduced the expression level of p-AKT, p-PI3K, and p-mTOR in a dose-dependent way. These results proposed that luteolin treatment blocked the PI3K signaling pathway [78]. Another study on melanoma found that luteolin affected the phosphorylation of AKT1 and PI3K. According to the study, immunofluorescence and immunoblotting assays showed that luteolin prevented the phosphorylation of these proteins. The study demonstrated that luteolin could inhibit the proliferation of melanoma cells and induce apoptosis through the reduction of MMP-2 and MMP-9 expressions, which is mediated by the PI3K/AKT pathway. This suggests that luteolin may have potential as a therapeutic agent in the treatment of melanoma. [79]. The PI3K/AKT/mTOR signaling pathway has been found to play a vital role in regulating the migration and invasion of cancer cells. Luteolin treatment decreased the protein levels of p-AKT as well as p-mTOR in a concentration-dependent way. This suggests that luteolin may be able to inhibit the PI3K/AKT/mTOR signaling pathway [80].

### 3.8. Wnt/β-Catenin Signaling Pathway

The Wnt/β-catenin pathway is a crucial regulator of various physiological processes such as embryonic development, adult tissue homeostasis, stem cell maintenance, wound healing, and more. It controls cell fate, differentiation, polarity, apoptosis, and migration in the body [81]. Recent studies have indicated that the dysregulation of the Wnt/β-catenin pathway is associated with the development and progression of various malignancies [82,83]. Pandurangan Ashokkumar (2011) reported that luteolin has been found to inhibit colon carcinogenesis by reducing Azoxymethane-induced cell proliferation through its involvement in key components of the Wnt signaling pathway such as β-catenin, cyclin D1, and Glycogen synthase kinase (GSK)-3β [84]. Luteolin is protective by inhibiting Azoxymethane-induced cellular proliferation, possibly through its involvement in key components of the Wnt signaling pathway, such as β-catenin, GSK-3β, and cyclin D1 [84]. The study was performed to examine the effects of luteolin on Wnt signaling. According to the results of the study, it was noticed that the administration of luteolin led to a noteworthy decrease in the mRNA levels of the target genes (C-Myc as well as Cyclin D1) of Wnt signaling in both primary and PC-3 spheres. This proposes that luteolin can inhibit Wnt signaling. Moreover, the results showed that luteolin suggestively decreased the transcriptional activity of β-catenin, which established that luteolin suppresses Wnt signaling in prostate cancer cells. Luteolin has been found to inhibit Wnt signaling in prostate cancer cells through the transcriptional upregulation of FZD6. This upregulation of FZD6 leads to the suppression of prostate cancer stemness [85].

### 3.9. Telomerase

Telomeres are specialized structures at the end of chromosomes that are made up of repetitive DNA sequences and proteins. Specifically, they consist of tandem DNA repeats (TTAGGG)n and have an average length of around 10 kilobases [86] and a core six-member protein complex [87,88] that plays a crucial role in protecting and regulating the ends of chromosomes. Many human cancers exhibit high levels of telomerase, even though telomerase is absent in most normal somatic tissues [89,90]. The levels of hTERT were examined to determine whether luteolin might have any correlative or mechanistic potential on breast cancer cells. The study’s results indicate that after 24 h of treatment with luteolin at concentrations of 10 and 30 µM, the mRNA expression levels of hTERT were significantly downregulated. Interestingly, treatment with luteolin for 48 h at similar concentrations resulted in the downregulation of hTERT mRNA in MDA-MB-231 cells. Furthermore, the study found that luteolin treatment caused a similar change in hTERT protein expression. These findings suggest that luteolin may inhibit telomerase activity in breast cancer cells by downregulating hTERT expression. The study also delved into the possibility that luteolin downregulated hTERT through the c-Myc pathway. It is worth noting that the study found that treatment with luteolin resulted in the downregulation of c-Myc expression in a time- and dose-dependent manner [62].

### 3.10. Activator Protein 1 (*AP*-*1*)

AP-1, a transcription factor, has been shown to regulate a wide range of cellular processes, including proliferation, differentiation, and apoptosis [91,92]. The effectiveness of luteolin in regulating interleukin-1β-induced production of MMPs and cytokines in a human synovial cell line, SW982, was examined. The study found that treatment with luteolin at 1 or 10 microM concentrations significantly inhibited the production of MMPs and cytokines induced by IL-1β. Furthermore, the study found that luteolin treatment prevented the activation of activator protein-1 (AP-1) and nuclear factor-κB (NF-κB) induced by IL-1β. These results advise that luteolin reduces the production of cytokines and MMPs in SW982 cells by preventing MAPKs and transcription factors (AP-1 and NF-κB) [93]. A study focused on breast cancer found that luteolin 8-*C*-β-fucopyranoside (LU8C-FP) was able to suppress the secretion and mRNA expression of IL-8 and MMP-9 induced by TPA treatment. This effect was found to be mediated by the down-regulation of nuclear AP-1 and NF-κB and the inhibition of the MAPK signaling pathway. The study has suggested that LU8C-FP may be able to suppress the invasion of breast cancer cells via the ERK/AP-1 and ERK/NF-κB signaling cascades. These findings propose that LU8C-FP may have potential therapeutic applications in breast cancer treatment [94].

### 3.11. Cell and Growth Factors Receptors

**Figure 5:**
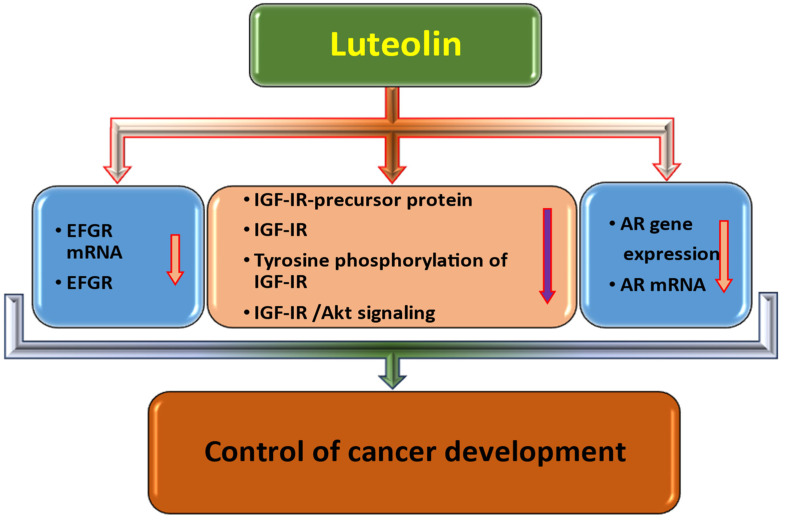
Role of luteolin in inhibition of cell receptor/growth factor receptor. The downward pointing arrow signifies downregulation.

Luteolin has been found to possess inhibitory effects on cell and growth factor receptor activities and their downstream effector molecules. As a result, various cellular processes that contribute to the progression and development of cancer cells are inhibited. Luteolin plays a significant role in the regulation of cell and growth factor receptors (Figure 5). Here discussed as:

i. Epidermal Growth Factor Receptor (EGFR)

The epidermal growth factor receptor (EGFR) is an essential member of the ErbB family of receptor tyrosine kinases (RTKs) and plays a vital role in various physiological processes of epithelial cells [95]. Interestingly, while mutated EGFRs found in tumors are enzymatically active and have transforming properties, their tyrosine phosphorylation status is significantly lower when compared to the ligand-activated wild-type receptors [96]. Altered expression of EFGR is found in cancers. The inhibitory potential of luteolin on MDA-MB-231 estrogen receptor (ER) negative breast tumor growth was examined in vitro and in vivo. Luteolin has a suppressive effect on H-thymidine incorporation, which could potentially lead to inhibition of cell growth. Specifically, the impact of luteolin on MDA-MB-231 cells that overexpress EGFR has been examined. Treatment with luteolin resulted in a dose-dependent reduction in the expression of EGFR mRNA. In addition to its impact on EGFR mRNA expression, luteolin inhibited EGF-induced autophosphorylation of EGFR. Based on these findings, it seems that luteolin is effective at suppressing the growth of MDA-MB-231 ER-negative breast cancer cells and that its potential as an anticancer agent may be partially due to its ability to inhibit EGFR-mediated cell survival [97]. A recent study explored how luteolin can inhibit the growth of breast cancer cells induced by epidermal growth factor. According to the survey, luteolin could impede the growth of MCF-7 cells triggered by EGF [98].

ii. Androgen Receptor

Androgen receptor (AR) is a steroid hormone receptor with a molecular weight of 110 kDa and is part of the family of steroid hormone nuclear receptors. It is also noted that the gene encoding for AR is also located on the X chromosome (Xq11–Xq12) [99]. Abnormal AR signaling contributes to different human diseases, including androgen insensitivity syndrome and prostate cancer [100]. Based on results from immunoblot assays and enzyme-linked immunosorbent assays, it has been observed that treatment with luteolin led to an upregulation of prostate-derived Ets factor while simultaneously downregulating AR gene expression. This ultimately resulted in a decrease in the expression of the PSA gene in LNCaP cells [101]. Studies have shown that luteolin significantly inhibits the proliferation and metastasis of androgen receptor-positive TNBC. In addition to this, when luteolin was combined with inhibitors of AKT/mTOR, it was found to have a synergistic effect in repressing androgen receptor-positive TNBC cell proliferation and metastasis [102]. Another study based on prostate cancer reported that luteolin has been found to have a dose- and time-dependent effect on the suppression of intracellular and secreted PSA levels, as well as the repression of AR mRNA and protein expression. Additionally, luteolin was observed to reduce the association between the androgen receptor and heat-shock protein 90, ultimately leading to AR degradation through a proteasome-mediated pathway independent of ligand presence [103].

iii. Insulin-Like Growth Factors (IGFs)

Insulin-like growth factors (IGFs) are polypeptides that stimulate the growth of a variety of mammalian cells [104]. At varying concentrations of 0–60 μmol/L, luteolin has a dose-dependent effect on the reduction of IGF-II secretion of HT-29 cells. In addition, luteolin was found to reduce the levels of the IGF-IR precursor protein and IGF-IR transcripts. Moreover, luteolin has also been found to reduce the IGF-I-induced tyrosine phosphorylation of IGF-IR and the association of p85 with IGF-IR. The results establish that luteolin downregulates the activation of the ERK1/2 and PI3K/Akt pathways through a decrease in IGF-IR signaling in HT-29 cells [105]. The study found that luteolin was able to inhibit the proliferation of human breast cancer MCF-7 cells in a dose- and time-dependent manner. Additionally, it was observed that luteolin significantly reduced IGF-1R and Akt phosphorylation induced by IGF-1, while Erk1/2 phosphorylation remained unaffected [106]. Another study based on prostate cancer reported that luteolin inhibited prostate cancer PC-3 tumor growth. Immunoblotting of the tumor tissues displayed that luteolin inhibited IGF-1R/AKT signaling [107].

### 3.12. Notch Signaling Cascade

It has been widely recognized that the Notch signaling cascade plays a vital role in various biological processes such as cell proliferation, differentiation, development, and homeostasis [108]. Altered Notch signaling is noticed in cancers [109]. Furthermore, high expression of Notch receptors, as well as ligands, was found to be linked with poor survival in breast cancer patients [110,111]. According to a study by Da-Wei Sun et al., in 2015, luteolin has been found to have a significant impact on preventing breast-cancer-cell survival, tube formation, and cell cycle, and luteolin has been found to affect the expression of Notch signaling-related proteins and mRNAs. The study also found that luteolin regulates miRNAs. Luteolin may inhibit Notch signaling by regulating miRNAs. Moreover, the downregulation of Notch-1, introduced by siRNA transfection, inhibited cancer-cell survival, migration ability, and HUVEC tube formations. Interestingly, the suppressive effects of Notch-1 downregulation were even more pronounced when 25 µmol/L of luteolin was added to Notch-1-silenced MDA-MB-231 cells. Notch-1 ICD cDNA was transfected to MDA-MB-231 cells and positively impacted the overexpression of Notch-1, which promoted cell survival and invasion. It appears that Notch-1 upregulation could also counteract luteolin’s inhibiting effect on cell growth and migration. However, the study also suggests that the effect of miRNAs on the Notch pathway may be either luteolin-dependent or luteolin-independent [112]. A pioneer study based on gastric cancer reported that luteolin significantly inhibited cell proliferation, invasion, and migration in a dose-dependent and time-dependent manner. It appears that luteolin prevented Notch-1 signaling, and downregulation of Notch-1 showed similar effects to luteolin treatment on cell proliferation, migration, and apoptosis. Notch-1 knockdown caused the promotion of cell apoptosis and reversed EMT in cancer cells. However, overexpression of Notch-1 recovered EMT in Hs-746T after luteolin treatment and raised AKT phosphorylation. Notch-1 overexpression partially reversed the inhibiting potential on cell migration by treatment of luteolin. These findings recommend that luteolin treatment suppressed this cancer progression via inhibiting Notch signaling. The finding revealed that luteolin treatment caused a decrease in β-catenin levels. It was also noticed that cyclin-D1, Notch-1, and Hes-1 were downregulated after luteolin treatment, demonstrating that luteolin prevented this cancer progression via suppressing Notch signaling [113].

### 3.13. Mitogen-Activated Protein Kinase (MAPK) Pathway

It is well known that MAPK cascades are essential for regulating cellular processes such as cell proliferation, differentiation, and responses to stress [114,115,116]. The gastric cancer-based study found that luteolin suppressed the phosphorylation of extracellular signal-controlled kinase in the MAPK signaling pathway. Luteolin decreased the phosphorylation of AKT, PI3K, and the mechanistic target of rapamycin in the PI3K signaling pathway [78]. The study established that luteolin suppressed cell proliferation, blocked the cell cycle, and induced DNA damage and apoptosis progression in CRC cells by mediating the MAPK pathway. Moreover, the potential role of luteolin on the MAPK signaling pathway was examined. The finding based on Western blot analysis reported that the levels of MAPK pathway-related proteins, such as ERK1/2 and MEK1, were not meaningfully altered, while the levels of p-ERK1/2 and p-MEK1 were decreased in a dose-dependent way in luteolin-treated HCT-116 cells. Additionally, the combination of luteolin (40 µM) and selumetinib reduced the phosphorylation levels of ERK1/2 and MEK1 as compared with selumetinib or luteolin only and decreased the level of the DNA repair protein XRCC1. Overall, luteolin shows a suppressive role in the behavior of colorectal cancer cells via inhibiting the MAPK signaling pathway [117]. The effect of luteolin on the MAPK pathway in glioma (LN229 and U251) cells was investigated. The study initially looked at JNK, P-JNK, ERK, P-ERK, p38, P-p38, Bax, and Bcl-2 expression levels in LN229 cells. It was noticed that upon treatment with luteolin, the expression levels of P-ERK increased, but the expression levels of ERK remained unchanged. The expression of P-JNK was enhanced in the luteolin-treated group compared to the control group. The results demonstrated that luteolin treatment led to the activation and phosphorylation of p38, JNK, and ERK. Based on these findings, it was suggested that luteolin could induce apoptosis by activating the MAPK pathway and death receptor pathway, thereby achieving an antitumor effect in human glioblastoma cells [44].

### 3.14. Invasion and Metastasis

According to recent research, proteolytic enzymes like matrix metalloproteinases (MMPs) can degrade both basal membrane (BM) and extracellular matrix (ECM) components. This degradation is responsible for the destruction of the peritumoral matrix, which enables an invasive growth pattern [118]. Recent studies have found that luteolin can inhibit the proliferation of MCF-7 cells in a dose- and time-dependent manner. Treatment with luteolin at 60 μmol/L resulted in a decreased migration rate of MCF-7 cells compared to the control group. Additionally, when MCF-7 cells were treated with luteolin at 60 μmol/L for 48 h, the expression of MMP-2 and AEG-1 was reduced by 85.70% and 82.34%, respectively, when compared to the control group. These findings suggest that luteolin could potentially be a therapeutic agent for cancer treatment. Additionally, luteolin showed anti-angiogenesis of chick chorioallantoic membrane and anti-invasive potential on breast cancer cells, and down-regulated the expression of MMP-2 and AEG-1 [119]. A vital study finding reported that the presence of luteolin prevented the effects of lipopolysaccharides (LPS). M1 polarization enhanced the proliferation rate, migration, invasion capability, and phosphorylation of STAT3 in colon cancer cells. Luteolin showed an important role in the inhibition of these effects via reducing M1 polarization. According to the study, IL-6 promotes cell proliferation, enhances migration and invasion, and increases STAT3 phosphorylation. Conversely, the anti-IL-6 antibody had the opposite effect. Furthermore, IL-6 promoted LPS-induced M1 polarization, whereas the anti-IL-6 antibody increased the decrease in luteolin-initiated M1 polarization. The finding concluded that luteolin suppresses the growth and migration/invasion potential of colon cancer cells. This was achieved by inhibiting the IL-6/STAT3 signaling pathway [120]. A recent study has found that luteolin treatment can significantly impact the migratory potential of certain types of cancer cells. Specifically, the study found that luteolin-treated HT-29 and SW620 cells significantly decreased cell migration and invasion. Further, research has shown that the expression of certain matrix metalloproteinases (MMPs) can be strongly associated with tumor migration and invasion in human colorectal cancer. Luteolin may have an impact on the expression of certain matrix metalloproteinases (MMPs) that are involved in tumor migration and invasion. According to recent research, luteolin was found to visibly downregulate the expressions of MMP-2, MMP-3, MMP-9, and MMP-16. However, interestingly, despite these in vitro results, consumption of luteolin did not appear to affect tumor growth in vivo, as no significant differences were observed in tumor volume or weight [121].

**Table 2:** Anti-cancerous potential of luteolin through modulation of numerous cell-signaling pathways. The upward pointing arrow signifies upregulation and downward pointing arrow signifies downregulation.

Signaling Pathways	Cancer Types	Study Types	Mechanism	The Outcome of the Study	Refs.
Inflammation	Colon cancer	In vitro and in vivo	IL-6, interferon-β, TNF-α, and IL-1 β ↓	Luteolin may be able to alleviate the growth of certain types of cancer by suppressing inflammatory processes in the body.	[23]
Angiogenesis	Prostate cancer		Angiogenesis (VEGF) and metalloproteinase (MMP 2,9) ↓	Luteolin inhibited ex vivo angiogenesis. This study found that luteolin was able to reduce the volume and weight of tumors.	[33]
Apoptosis	Cervix cancer	In vitro	Apaf1, Bax, Bad & Caspases ↑ & Bcl 2 ↓	Luteolin treatment enhances early apoptotic cells. Increase in the amount of late apoptotic.	[42]
	Colon cancer	In vitro	Bax ↑ and Bcl2 ↓	Luteolin enhanced the expression of the apoptotic protein. It decreased the expression of the anti-apoptotic protein.	[43]
	Brain cancer	In vitro	Apoptosis induction	The percentages of early and late apoptotic cells increased with luteolin.	[44]
Autophagy	Ovarian	In vitro	Suppression of autophagy ↓	Luteolin suppresses autophagy.	[53]
	Lung cancer	In vitro	LC3 puncta and autophagy flux ↑	It also initiates both endoplasmic reticulum stress-related apoptosis and non-canonical autophagy.	[55]
Cell cycle	Colon cancer	In vitro	% of cells in G_1_ ↓ and G_2_/M phase ↑	Luteolin can promote G2/M arrest.	[61]
	Breast cancer	In vitro	Cell-cycle arrest in S-phase	It can induce dose-dependent cell-cycle arrest in the S-phase, which may lead to the suppression of cancer-cell growth.	[62]
STAT3	Gastric cancer	In vitro and in vivo	STAT3 ↓	Luteolin reduced the expression of STAT3 targeting gene and inhibited STAT3 phosphorylation. Luteolin downregulated STAT3 phosphorylation	[68]
	Skin cancer	In vitro and in vivo	STAT3 ↓	Luteolin inhibited phosphorylation of STAT3. In vivo studies have proven that luteolin restrained cancer growth and Src/STAT3 signaling.	[69]
PI3K/AKT/mTOR	Breast cancer	In vitro	PI3K/AKT/mTOR ↓	Luteolin has the potential to reduce the levels of activated PI3K/AKT/mTOR signaling pathways.	[76]
	Gastric cancer	In vitro	PI3K/AKT ↓	Luteolin suppressed phosphorylation of AKT/PI3K.	[78]
	Skin cancer	In vitro and in vivo	PI3K/AKT ↓	Luteolin prevented the proliferation as well as induced the apoptosis by decreasing the expressions of MMP-2 -9 via the PI3K/AKT pathway.	[79]
Wnt signaling	Prostate cancer	In vitro	Wnt ↓	Luteolin decreased the mRNA levels of the target genes (Cyclin D1 and C-Myc) of Wnt signaling.	[85]
Telomerase	Breast cancer	In vitro	Telomerase ↓	Luteolin has some promising abilities in suppressing the expression of human telomerase reverse transcriptase by stopping the phosphorylation of the nuclear factor-κB inhibitor α and its target gene.	[62]
Transcription factors AP-1	Synovial sarcoma		AP-1 ↓	Luteolin decreases the production of cytokines in cancer cells by preventing MAPKs and transcription factors AP-1.	[93]
Epidermal growth factor receptor	Breast cancer	In vivo and in vitro	EGFR ↓	Luteolin has the ability to efficiently suppress cancer-cell growth, and its anticancer activity may be partially derived from inhibitory effects on EGFR-mediated cell survival.	[97]
		In vitro	EGFR ↓	Luteolin has been found to have the ability to inhibit EGF-induced activities of the EGFR signaling pathway.	[98]
Notch signaling	Breast cancer	In vitro	Notch ↓	Luteolin inhibited cell cycle and the expression of Notch signaling-linked proteins and mRNAs.	[112]
	Gastric cancer	In vitro	Notch ↓	Notch-1 signaling was inhibited by luteolin.	[113]
Mitogen-activated protein kinase (MAPK)	Gastric cancer	In vitro	MAPK ↓	Luteolin employs a dual prevention on both the PI3K and MAPK signaling pathways.	[78]
	Colorectal cancer	In vitro	MAPK ↓	This compound may have an inhibitory task in suppressing the MAPK signaling pathway.	[117]

## 4. Luteolin Shows the Ability to Target microRNAs (miRs) in Cancer

**Figure 6:**
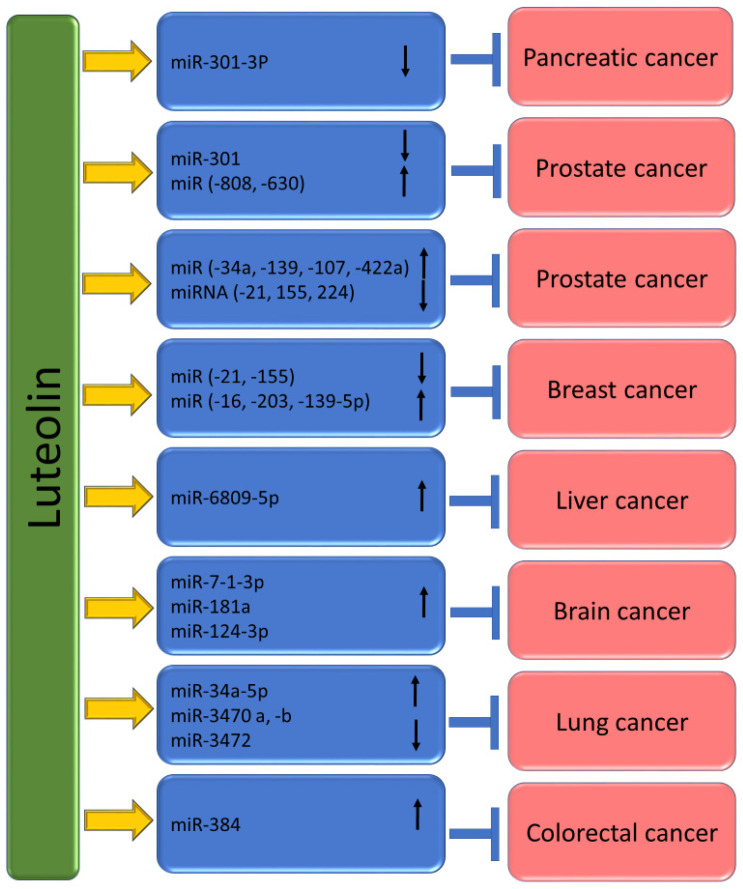
Luteolin plays a role in cancer management through the regulation of microRNAs. The upward pointing arrow signifies upregulation and the downward pointing arrow signifies downregulation. The block arrow signifies the inhibition.

MicroRNAs are undoubtedly small, non-coding RNA molecules that suppress the expression of genes by inhibiting protein translation as well as encouraging mRNA cleavage [122]. It is interesting to know that between 200 and 255 genes encode miRNAs [123]. These small RNA molecules play important roles in regulating gene expression and have been implicated in various biological processes. Interestingly, miRNAs are transcribed from DNA sequences into primary miRNAs, which are then processed into precursor miRNAs and mature miRNAs [124]. Furthermore, miRNAs have been revealed to control essential features of cancer, for example, maintaining proliferative signals, resisting cell death, evading growth inhibition, angiogenesis, immortalizing replication and invasion, as well as metastasis [125,126]. It is interesting to know that studies indicate that natural compounds can be used to modulate miRNA expression to promote cancer-fighting action [127,128]. It is interesting to know that curcumin, a natural compound found in turmeric, has been found to use miRNA to inhibit the growth of cancer cells [129]. The anti-cancerous role of luteolin is confirmed through the regulation of microRNAs (miRs) (Table 3 and Figure 6).

Hao Wu et al., based on gastric cancer cells, produced a report stating that micro-RNA (miR)-34a has direct control over Bcl-2 and that overexpression of miR-34a can reduce the levels of Bcl-2 protein. Interestingly, luteolin can downregulate the expression of Bcl-2 while simultaneously upregulating the expression of miR-34a [130]. According to microarray analysis of H460 tumor xenografts, it was found that the luteolin high-dose group (200 mg/kg) had a significant upregulation of 20 miRNAs, including miR-34a-5p, and a suggestive downregulation of four miRNAs compared to the control group and it has been reported that the expression of miR-34a-5p was found to be upregulated in a dose-dependent manner following luteolin treatment in vivo [133]. To examine the potential mechanisms by which luteolin initiated apoptosis of prostate cancer cells, the study recognized the miRNAs modulated by luteolin using miRNA microarray. The results of the miRNA microarray analysis showed that luteolin treatment led to a significant decrease in the expression of miR-301 by a factor of 5.39, indicating that this miRNA was downregulated in response to luteolin treatment. Pathological section samples were obtained from 54 PCa patients in this study. The miR-301 expression was examined using ISH and qRT-PCR methods. According to the findings, the expression of miR-301 showed a correlation with distant metastasis, extracapsular extension, seminal vesicle invasion, Gleason score, and stage. However, no significant differences were observed in other clinical features like age, prostate-specific antigen (PSA), and surgical margin between individuals with high as well as low miR-301 expressions. The Kaplan–Meier survival analysis revealed that patients with high miR-301 expression tended to show shorter overall survival (OS) as compared to those with low miR-301 expression. Moreover, to investigate the impact of miR-301 on the functional responses induced by luteolin, experiments were conducted in which PC3 and LNCaP cells were treated with miR-301-MIMIC or control NC in the presence of luteolin. The results showed that when miR-301-MIMIC was combined with luteolin, it significantly counteracted the effect of luteolin on cell viability, in comparison to both luteolin alone and the normal control group [134].

Another experiment based on pancreatic ductal adenocarcinoma reported that the levels of miR-301-3p were down-regulated in PANC-1 cells exposed to luteolin, which prevents the growth of PANC-1 cells as well as sensitizes cells to TRAIL. Further, the study aimed to determine whether the levels of miRNA-301-3p can be influenced by luteolin treatment. As per the findings, luteolin treatment reduced the levels of primary transcripts of miRNA-301a and miRNA-301b. Furthermore, a decrease in the levels of mature miRNA-301-3p (including miRNA-301a-3p and miRNA-301b-3p) was observed in the cells exposed to luteolin [135].

To examine the molecular pathway of luteolin, expression levels of miR-16, -21, and -34a were assessed in luteolin-treated MCF-7 cells. The result demonstrated that treatment with luteolin exhibited an approximately 2.4-fold increase in both miR-16 and -34a expression, and a reduction in the expression of miR-21, 48 h post-treatment [136].

A study based on breast cancer reported that MiR-181a, miR-224, miR-139-5p, and miR-246 expression levels in both MCF-7 and MDA-MB-231 cells were meaningfully increased after luteolin treatment, whereas miR-155 level reduced. Moreover, MiR-34a expression only enhanced in luteolin-treated MDA-MB-231 cells. The results advocate that luteolin distinctively altered miRNA levels in breast cancer cells. After Notch-1 siRNA transfection, MDA-MB-231 cells showed enhanced miR-34a, miR-181a, miR-224, miR-139-5p, and miR-246 expression levels, but showed decreased miR-155 levels. These findings were similar to MDA-MB-231 cells treated with luteolin [112].

A pioneer study based on breast cancer revealed that luteolin elevates the miR-203 level. In addition, luteolin’s anti-tumor potential was partly eliminated by miR-203 silence. Luteolin meaningfully reduced breast-cancer-cell growth as well as EMT. This compound employed its anti-tumor property, probably involving the raised expression of miR-203 as well as the inhibited Ras/Raf/MEK/ERK signaling [137].

The study has found that both luteolin as well as silibinin can stimulate the expression of miR-7-1-3p. The research shows that the levels of expression of three miRs, including miR-7-1-3p, miR-181a, and miR-9, were evaluated in glioblastoma U87MG and T98G cells. These cells were pretreated with RAPA and treated with luteolin and/or silibinin. All the tumor-suppressor miRs were upregulated after the treatment with luteolin and/or silibinin, as compared to RAPA-pretreated cells. This finding suggests that luteolin and silibinin may have potential therapeutic benefits in the treatment of glioblastoma. The study also found that the ectopic overexpression of miR-7-1-3p, combined with therapy using luteolin and silibinin, resulted in a significant decrease in the growth of RAPA-pretreated U87MG and T98G xenografts in athymic nude mice. Molecular studies conducted on glioblastoma xenografts have revealed that the overexpression of miR-7-1-3p enhances the effectiveness of the combination of luteolin and silibinin in monitoring the in vivo growth of human glioblastoma harboring wild-type p53 or mutant p53 [138]. It appears that in LN229 cells, the expression levels of four miRNAs were analyzed after luteolin treatment. Results showed that the only miRNA that showed significant upregulation in the luteolin-treated group compared to the control group was miR-124-3p, while the other three miRNAs remained unchanged in both LN229 and U251 cells. The relationship between miR-124-3p and the effect of luteolin on cell proliferation was studied. It was reported that overexpression of miR-124-3p could significantly increase cellular cytotoxicity, while downregulation of miR-124-3p could decrease cellular cytotoxicity in LN229 cells [44]. It has been reported that transfecting miR-8080 can lead to a decrease in the expression of AR-V7 and can also trigger apoptosis in 22Rv1 cells. In addition, it has been observed that knocking down miR-8080 can reverse the effects of luteolin, leading to an increase in the expression of AR-V7 and cell growth in 22Rv1 cells [139]. The colorectal cancer-based study reported that luteolin has been shown to upregulate the expression of miR-384 [121]. Moreover, luteolin shows anticancer potential against colorectal cancer (CRC) cells by modulating PTN via miR-384 expression suggesting that PTN may serve as a valuable candidate for therapeutic applications in CRC treatment [121]. It appears that treatment of prostate cancer cells by luteolin and/or gefitinib caused increased expression of miR-630 as well as miR-5703 [140]. The study suggests that luteolin was found to significantly prevent the growth of HCC (hepatocellular carcinoma) and induce apoptosis (cell death) and cell-cycle arrest. The study also discovered that luteolin can upregulate the expression of miR-6809-5p and that overexpression of miR-6809-5p can lead to the suppression of HCC cell growth. The study conducted loss-of-function experiments to investigate whether the upregulated miRNAs are responsible for the growth-suppressive activity of luteolin. Transfection with anti-miR-6809-5p meaningfully blocked the enhancement of miR-6809-5p by luteolin in Huh7 cells. Reduction of miR-6809-5p meaningfully reversed the inhibitory effect of luteolin on cell proliferation and colony formation [141].

## 5. The Anti-Cancer Activity of Luteolin in Comparison with Other Flavonoids/Similar Compounds

The anti-cancer potential of various types of flavonoids has been examined through comparative analysis based on their action on cancer cells. Here, the studies of different flavonoids, along with luteolin, in cancer management are summarized. This study is a report on the antiproliferative activities of various citrus flavonoids against tumor cell lines. According to the report, seven flavonoids were effective against the tumor cell lines. The flavonoids were ranked in order of potency, with luteolin being the most potent and 3,3′,4′,5,6,7,8-heptamethoxyflavone being the least potent [142]. Another study was performed to examine the anti-proliferative activity of different related compounds. Results revealed that linalool had the most potent activity against carcinoma of the cervix, stomach, skin, lung, and bone among the compounds studied. Its IC50 values for these cancer types were 0.37 microg/mL, 14.1 microg/mL, 14.9 microg/mL, 21.5 microg/mL, and 21.7 microg/mL, respectively. The flavonoids also showed promising anti-cancer activity, with luteolin and baicalein being the most potent against certain types of cancer. Luteolin had the strongest activity against carcinoma of the stomach (IC50: 7.1 microg/mL), cervix (IC50: 7.7 microg/mL), lung (IC50: 11.7 microg/mL), and bladder (IC50: 19.5 microg/mL). On the other hand, baicalein exhibited the strongest anti-proliferative activity against carcinoma of the cervix (IC50: 9.8 microg/mL), stomach (IC50: 16.1 microg/mL), and skin (IC50: 19.5 microg/mL) [143]. The role of eight flavonoids on cellular protein phosphorylation, tumor-cell proliferation, and matrix metalloproteinase secretion was examined. Luteolin and quercetin stood out as the most potent flavonoids among those examined. These two compounds significantly inhibited the proliferation of A431 cells, with IC50 values of 19 and 21 micronM, respectively. At a concentration of 20 micronM, both luteolin and quercetin were observed to significantly impact the phosphorylation levels of several cellular proteins in A431, including EGFR [144]. An interesting study compared the effects of different bioflavonoids on the growth of androgen-independent human prostatic tumor cells. It was found was that quercetin, kaempferol, and luteolin were the most effective in completely stopping the growth of cancer cells when treated with 100 microM. Genistein, apigenin, and myricetin were also effective but to a lesser degree, suppressing PC-3 proliferation by 73%, 70%, and 59%, respectively. However, naringenin and rutin were less effective and only inhibited growth by less than 25% [145].

A study was conducted to analyze the relationship between the consumption of five common dietary flavonoids and the risk of epithelial ovarian cancer. The study involved 66,940 women who participated in the Nurses’ Health Study. There was no strong link between the incidence of ovarian cancer and the total intake of the five common dietary flavonoids tested. However, there was a significant 40% decrease in ovarian cancer incidence for the highest versus lowest quintile of kaempferol intake and a significant 34% decrease in incidence for the highest versus lowest quintile of luteolin intake [146]. Another study based on human leukemia cells reported that apigenin and luteolin powerfully inhibited the growth of HL60 cells as well as induced morphological differentiation into granulocytes. Moreover, other flavones such as quercetin, galangin, naringenin, and kaempferol also show inhibition effects but do not induce morphological differentiation [147]. The study aimed to examine the impact of 23 different flavonoids on the proliferation of the HL-60 leukemia cell line. According to the study, the majority of the 23 flavonoids examined showed a significant inhibition of the proliferation of HL-60 cells. Furthermore, the inhibition effects were found to increase as the concentration of the flavonoids increased. The study also found that the intensity of the flavonoids’ effects varied, with some showing stronger inhibition than others. The flavonoids were arranged in order of strength, with 3,6-dihydroxyflavone, luteolin, geraldol, 2′-hydroxyflavanone, and apigenin being the most potent inhibitors, while naringenin, hesperetin, daidzein, 7-hydroxyflavone, and flavone were found to have the weakest effects [148]. The cytotoxic effects of luteolin and apigenin on two different cancer-cell lines, such as human chronic myelogenous erythroleukaemia and bladder carcinoma, were examined. The outcomes of an MTT assay showed that apigenin and luteolin could induce cytotoxicity in RT112 and K562 cells in a dose- and time-dependent manner. The cytotoxic potency of luteolin was higher than that of apigenin [149].

## 6. The Potential Role of Luteolin in Cancer Using Molecular Modeling Techniques

Computer-aided drug-design methods, which include virtual screening, molecular docking, and molecular-dynamics simulation approaches, allow researchers to competently analyze and assess potential drug candidates [150,151,152,153]. Moreover, computational chemistry has become an increasingly valuable tool in drug discovery. Using various computational approaches, medicinal chemists can explore a wide range of theoretical aspects of drug discovery [154]. Various computational approaches based on different flavonoids were used to recognize such compounds’ drug targets and pharmacological mechanisms. A study focused on finding new inhibitors of MEK2 from flavonoids by using various techniques such as virtual screening, pharmacokinetic prediction, molecular docking analyses, and molecular-dynamics simulations. Using molecular docking, the study screened a library of drug-like flavonoids, which had 1289 chemical compounds, against the MEK2 allosteric site. According to an in-silico study, ten flavonoids were proposed as potential inhibitors of the anticancer therapeutic target MEK2. These selected compounds exhibited good binding affinity and ligand interactions, indicating that they could be better inhibitors of MEK2 compared to the native one [155]. A study explored the potential anti-cancer properties of luteolin, a natural compound found in Tridax procumbens, on human lung cancer cells. This study conducted a computational study to investigate the structural characteristics of luteolin. Moreover, virtual screening was used to predict the drug-likeness of the molecule based on its ADMET properties. To summarize the in-silico study results, it looks like luteolin has emerged as a strong candidate for potentially inhibiting human lung cancer. Through computational analysis and virtual screening of ADMET properties, the researchers identified luteolin as a natural compound found in Tridax Procumbens that could serve as an effective inhibitor against lung cancer [156]. The study presented a simple and practical method of creating zinc oxide nanoparticles using luteolin obtained from Eclipta alba plant (L-ZnONPs). The research findings indicate that L-ZnONPs exhibit a higher level of anticancer activity against the MCF-7 breast cancer-cell line compared to luteolin or ZnO alone. Further analysis using in silico techniques revealed that the anti-breast cancer activity of L-ZnONPs was linked to the regulation of polo-like kinase 1 (PLK1) proteins [157]. Through the data mining of network pharmacology as well as bioinformatics analysis, it was found that luteolin can interfere with the occurrence as well as development of hepatocellular carcinoma via multi-gene, multi-target, and multi-pathway means. It is interesting to note that after visualization, the main core targets of mignonette in treating hepatocellular carcinoma are SRC, EGF, ESR1, AKT1, PI3KR1, AR, and CDK1. It is particularly noteworthy that SRC and ESR1 seem to have a significant effect on the survival curve of patients with hepatocellular carcinoma [158]. It was found that luteolin downregulated four genes (AP2B1, APP, GPNMB, and DLST) in breast-cancer-cell lines. These genes are associated with drug resistance, cell proliferation, macrophages, apoptosis, and HDAC inhibition. Moreover, it was found that luteolin-mediated differentially expressed genes (DEGs) were involved in T-cell leukemia virus 1 infection and differentiation, according to KEGG pathway analysis [159]. The study was conducted to investigate the potential drug target and curative mechanism of luteolin as a treatment for prostate cancer. The differential gene expression in this cancer case was determined via RNA sequencing. The study also utilized network pharmacology as well as molecular docking to recognize the drug targets and pharmacological mechanisms of luteolin. The study found the top 20 up- and downregulated gene expressions in patients with prostate cancer [160].

## 7. Safety, Pharmacokinetics and Nano-Delivery System of Luteolin in Cancer Treatment

Safety is of utmost importance for any compound for therapeutic purposes, in addition to bioavailability and efficacy. Flavonoids are generally safe for consumption and possess a high safety profile. Luteolin, a flavonoid, is a safe compound that does not cause adverse effects when taken orally or intravenously at specific doses. The LD50 for luteolin has been found to be 460 mg/kg based on the results, and it is worth noting that 100 mg/kg of luteolin did not cause any liver or kidney toxicity, which indicates a favorable safety profile for luteolin [161].

Even though there is the auspicious nutritional and health-beneficial potential of luteolin, luteolin shows low aqueous solubility, low intestinal absorption, and poor biodistribution. Metabolism, absorption, and excretion of luteolin have been described in several studies as, after oral administration of *C. morifolium* extract to rats, luteolin as well as its glycosides were rapidly absorbed and luteolin monoglucoside and luteolin monoglucuronide were noticed in the plasma. Their levels were maximum at 1 h after administration (0.76 ± 0.27 μM). Luteolin and luteolin monoglucoside are easily absorbed after the administration of *C. morifolium* flower extract. It appears that luteolin monoglucoside, luteolin, and luteolin monoglucuronide may circulate in humans [162]. K Shimoi et al. explored the intestinal absorption of luteolin and luteolin 7-*O*-β-glucoside in rats using HPLC. It appears that research has shown that luteolin is transformed into glucuronides as it passes through the intestinal mucosa and that luteolin 7-*O*-β-glucoside is absorbed after being broken down into luteolin. According to the research findings, free luteolin and its conjugates and methylated conjugates were detected in the plasma of rats after being dosed. This advises that some luteolin can escape the intestinal conjugation as well as the hepatic sulfation/methylation. It was observed that the concentration of plasma luteolin and its conjugates peaked at 15 min and 30 min, respectively, after dosing with luteolin in propylene glycol [163]. The study investigated luteolin’s glucuronidation and methylation pathways, focusing on the interplay of catechol-*O*-methyltransferases (COMTs) and UDP-glucuronosyltransferases (UGTs) both in vitro and in vivo. Using liquid chromatography-tandem mass spectrometry, a total of nine luteolin metabolites were observed in both rat plasma and bile during the study. Among the nine observed luteolin metabolites, luteolin-3′-glucuronide (Lut-3′-G) showed the highest systemic exposure during the study [164].

Luteolin glucosides, luteolin-7-*O*-[2-(β-d-apiosyl)-6-malonyl-β-d-glucoside]), and luteolin-7-*O*-[2-(β-d-apiosyl)-β-d-glucoside] were prepared from green pepper leaves as well as luteolin aglycone were orally administered to rats. Irrespective of the administered form of luteolin, it was found that luteolin glucuronides were primarily observed in both plasma and organs. As a result, luteolin aglycone, the most absorbed form of luteolin in rats, was given to humans orally [165].

Even though luteolin has auspicious nutritional and health-beneficial potential, it shows low aqueous solubility, low intestinal absorption, and poor biodistribution. Metabolism, absorption, and excretion of luteolin have been described in several studies, as after oral administration of *C. morifolium* extract to rats, luteolin as well as its glycosides were rapidly absorbed and luteolin monoglucoside and luteolin monoglucuronide were noticed in the plasma. Their levels were maximum at 1 h after administration (0.76 ± 0.27 μM). The results suggest that luteolin and luteolin monoglucoside are rapidly absorbed upon administration of *C. morifolium* flower extract and that luteolin monoglucoside, luteolin, and luteolin monoglucuronide may circulate in humans [162]. K Shimoi et al., experimented to examine the absorption of luteolin as well as luteolin 7-*O*-β-glucoside in rats using HPLC. Scientific research on luteolin has found that it can be converted to glucuronides when passing through the intestinal mucosa. Additionally, luteolin 7-*O*-β-glucoside can be absorbed after being hydrolyzed to luteolin. Rat plasma samples from dosing experiments showed the presence of free luteolin, its conjugates, as well as methylated conjugates. This advises that some luteolin can escape the intestinal conjugation as well as the hepatic sulfation/methylation. In addition, the plasma concentration of luteolin and its conjugates were found to peak at 15 and 30 min, respectively, after dosing with luteolin in propyleneglycol [163]. The investigation of luteolin’s glucuronidation and methylation pathways revealed that they are mediated by the interplay of catechol-*O*-methyl transferases (COMTs) and UDP-glucuronosyltransferases (UGTs), both in vitro and in vivo. A total of nine luteolin metabolites were observed in rat plasma and bile through liquid chromatography-tandem mass spectrometry. It seems that among the metabolites that were investigated, luteolin-3′-glucuronide (Lut-3′-G) had the highest systemic exposure. The metabolic disposition of luteolin is believed to involve two pathways that are mediated by the interplay of catechol-*O*-methyltransferases (COMTs) and UDP-glucuronosyltransferases [164]. Luteolin glucosides, luteolin-7-*O*-[2-(β-d-apiosyl)-6-malonyl-β-d-glucoside]), and luteolin-7-*O*-[2-(β-d-apiosyl)-β-d-glucoside] were prepared from green pepper leaves as well as luteolin aglycone were orally administered to rats. Irrespective of the administered luteolin form, luteolin glucuronides were chiefly observed in plasma and organs. Consequently, luteolin aglycone, the most absorbed form of luteolin in rats, was orally administered to humans [165]. The poor solubility of luteolin in water is a major hindrance to its efficacy in cancer treatment, as it results in poor bioavailability [166].

**Figure 7:**
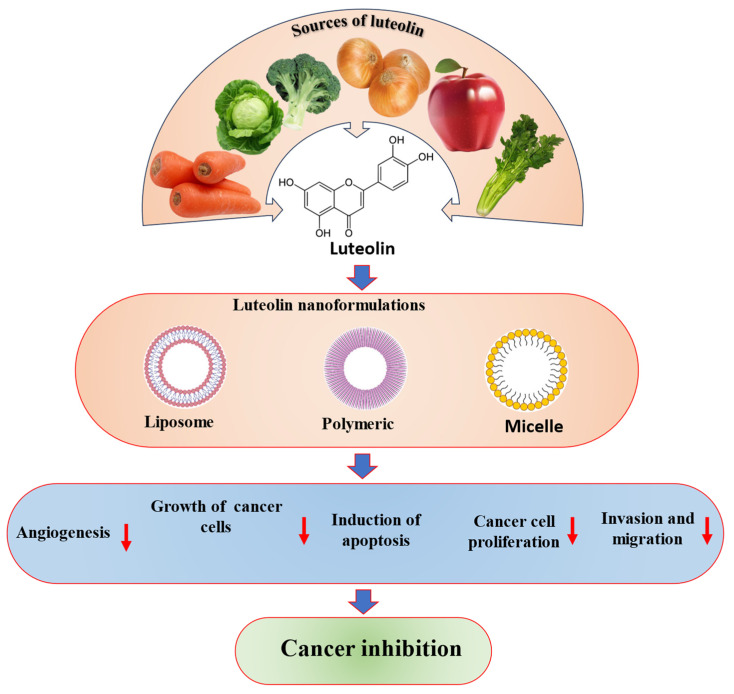
Nano-formulation of luteolin in cancer management. The upward pointing arrow signifies upregulation and the downward pointing arrow signifies downregulation.

Researchers have tried to use nano-delivery systems to improve the poor bioavailability of luteolin and enhance its efficacy (Figure 7 and Table 4). Numerous delivery systems have been formulated based on nanotechnologies to improve luteolin’s bioavailability, efficacies, and target ability. Luteolin-loaded Her-2-poly (lactic-co-glycolic acid) (PLGA) nanoparticles resulted in a significantly increased uptake of luteolin by SGC-7901 cells compared to non-targeted microspheres [167]. A recent study investigated the potential of transdermal elastic liposomes (LEL1–LEL12) loaded with luteolin (LUT) to control breast cancer. According to the study, the concentration of luteolin in both the standard form and the formulated transdermal elastic liposomes (LELs) affected the cell viability of MCF7, a breast cancer cell. Moreover, the formulation of luteolin has increased growth inhibitory effects in MCF7 cells. The data from the cell viability assay showed that the luteolin-containing formulation had a significantly increased impact in reducing the IC50 compared to standard luteolin [168].

The research indicates that L-ZnONPs (luteolin-loaded zinc oxide nanoparticles) may have a higher anticancer activity against a breast-cancer-cell line than either luteolin or zinc oxide alone [157,169]. Nano pyrosomes of luteolin were prepared to increase its bioavailability and improve passive aiming in breast cancer cells. Results revealed that co-treatment of cells with nanoparticles holding luteolin and doxorubicin caused the highest percentage of cell death in MDA-MB 231 cells. The study also found that luteolin-loaded nanoparticles were more effective in reducing Nrf2 (a gene that regulates oxidative stress) expression at the mRNA level in cells compared to luteolin on its own [169]. The study found that Luteolin/MPEG-PCL micelles were more effective in inducing cytotoxicity and apoptosis in C6 and U87 cells compared to free luteolin in vitro. Additionally, these micelles were found to cause more apoptosis in glioma cells and inhibit neovascularization in tumor tissues compared to free luteolin [170].

## 8. Conclusions and Future Prospects

Cancer is undoubtedly a complex and severe disease that affects many people worldwide. A combination of various factors causes it and can lead to significant mortality. While there have been significant advancements in treatment methods such as surgery, chemotherapy, and radiotherapy, there is still a need for more effective therapies. Because of the adverse effects of current cancer treatment modules, there is a clear need to develop new therapies that are safe, effective, and affordable. In this regard, recent research has revealed that luteolin, a polyphenolic flavonoid chiefly found in vegetables and fruits, may hold promise as a potential remedy for cancer treatment by inhibiting inflammation, angiogenesis, oxidative stress, and cell proliferation, and inducing autophagy and induction, cell-cycle arrest, and modulation of various other cell-signaling pathways. Further, the anticancer potential of this compound is established through its upregulation and downregulation of different micro-RNA. Despite the anti-cancerous potential of luteolin, its usefulness is restricted due to numerous factors such as low solubility, poor absorption, as well as rapid metabolism. To overcome such limitations, targeted drug-delivery systems like nanotechnology are in practice to enhance bioavailability and activity, but more studies or explanations are required to address these limitations. The combination treatment of luteolin with anticancer drugs showed greater efficiency in killing tumor cells, reduced cancer-cell viability, induced apoptosis, and modulated the cell signaling pathways compared to anticancer drugs alone. However, more combinational treatment with cancer drugs is needed to avail its better use in cancer treatment. Additionally, derivatives of luteolin must be synthesized to explore their anticancer activity. The detailed mechanism of action and the safety level of this compound is required to inspect its potential role in disease management. Additionally, in vivo and in vitro studies have confirmed the role of luteolin in cancer management through the modulation of cell signaling pathways. Still, the lack of robust clinical trials limits its utility. However, it is important to note that further clinical trials are needed to explore the potential of this compound as a full anticancer treatment in humans. The extensive scientific evidence compiled in this review will help the researchers to explore a hopeful remedy against different pathogenesis, including cancer.

## Figures and Tables

**Table 1:** Sources of luteolin with quantity (mg/kg) [14].

Vegetables/Fruits	Quantity (mg/kg)
Broccoli	74.5
Green chili	33.0
Bird chili	1035.0
Onion leaves	391.0
Belimbi fruit	202.0
Belimbi leaves	464.5
French bean	11.0
Carrot	37.5
White radish	9.0
Local celery	80.5
Limau purut leaves	30.5
Dried asam gelugur	107.5

**Table 3:** Luteolin shows a role in cancer management through the down and upregulation of microRNAs (miRs).

Cancer Types	Study Types	Regulation of microRNAs	Outcomes	Refs.
Gastric	BGC-823 and SGC-7901	Upregulation of miR-34a	MiR-34a overexpression decreased Bcl-2 expression and induced apoptosis. Luteolin downregulates Bcl-2 expression and upregulates miR-34a expression.	[130]
	MKN45 or BGC823	Upregulated miR-107, miR-34a and miR-139	Increased after different doses of luteolin treatment.	[131]
	MKN45 or BGC823	Upregulated miR-34a and miR-422a	Increased after different doses of luteolin treatment.	[131]
	MKN45 or BGC823	Downregulated miR-21, miR-155	Decreased after different doses of luteolin treatment.	[131]
	MKN45 or BGC823	Downregulated miR-224, miR-340	Decreased after different doses of luteolin treatment.	[131]
	AGS, BGC823 and SGC7901	MiR-34a upregulated	MiR-34a upregulated in cancer cells induced by luteolin. MiR-34a increases the susceptibility of cancer cells to luteolin.	[132]
Lung	H460 xenografts mice model	Upregulated 20 miRNAs, including miR-34a-5p and Downregulated miR-3470a, miR-3470b, miR-3472 and miR-3105-5p	MiRNA expression was changed through upregulation and downregulation in response to luteolin in H460 tumor xenografts.	[133]
Prostate	PC3 and LNCaP	Downregulated miR-301	MiR-301 was found to be downregulated after treatment with luteolin.	[134]
Pancreas	PANC-1	Down-regulates miRNA-301-3p	MiR-301-3p downregulated in cancer cells exposed to luteolin.	[135]
Breast	MCF-7	Downregulated the expression of miR-21 and upregulated that of miR-16	Luteolin meaningfully upregulated the expression of miR-16 and downregulated the expression of miR-21 and -34a.	[136]
	MDA-MB-231 and MCF-7	Upregulated miR-139-5p, MiR-181a, miR-224 and miR-246 Downregulated miR-155	In cancer cells, luteolin treatment increased the expression levels of miR-139-5p, miR-181a, miR-224, and miR-246, while miR-155 expression decreased.	[112]
	MDA-MB-453 and MCF-7	Upregulated miR-203	Luteolin’s anti-tumor properties may be related to its ability to increase the expression of miR-203 and inhibit the Ras/Raf/MEK/ERK signaling pathway.	[137]
Brain	U87MG, T98G cells and U87MG and T98G xenografts nude mice	Upregulated miR-7-1-3p, miR-181a and miR-9	Tumor suppressor miRs were upregulated by treatment with luteolin.	[138]
	U251	Upregulation miR-124-3p	Luteolin enhanced the expression levels of miR-124-3p.	[44]
Prostate	22Rv1 and VCaP	Upregulation of miR-8080	Luteolin prompted an increase in the expression of miR-8080.	[139]
Colorectal	HT-29 and SW480	Upregulated miR-384	Luteolin has been shown to upregulate the expression of miR-384.	[121]
Prostate	PC-3	Upregulation of miR-630 and miR-5703	It appears that treatment of prostate cancer cells by luteolin and/or gefitinib caused increased expression of miR-630 as well as miR-5703.	[140]
Liver	Huh7	Upregulated miR-6809-5p	It appears that luteolin can upregulate miR-6809-5p.	[141]

**Table 4:** Luteolin-based nano-formulation and its effects on cancer cells.

Nano-Formulation	Cancer Type	Findings	Refs.
Luteolin-loaded Her-2-poly (lactic-co-glycolic acid)	Gastric	Her-2-NPs directed to meaningfully increased uptake of luteolin by cancer cells. This nanoformulation prevented the proliferation of gastric cancer cells.	[167]
Luteolin-loaded elastic liposomes	Breast	OLEL1 exhibited maximized inhibition as compared with DS.	[168]
Luteolin-fabricated ZnO nanostructures	Breast	L-ZnONP treatment showed better anti-breast cancer potential against cancer cells.	[157]
Luteolin-loaded phytosomes	Breast	It was found that nanoparticles loaded with luteolin were more effective in reducing Nrf2 gene expression at the mRNA level in cells compared to luteolin alone. The expression of downstream genes for Nrf2, including Ho1 and MDR1, were significantly decreased.	[169]
Luteolin-loaded MPEG-PCL (luteolin/MPEG-PCL) micelles	Brain	Luteolin/MPEG-PCL micelles showed higher cytotoxicity and induced a greater percentage of apoptosis.	[170]
